# COVID: The Missing Trigger to Start a Remote FIT Course

**DOI:** 10.3389/fcdhc.2022.834082

**Published:** 2022-02-17

**Authors:** Marcelo dos Santos Mamed, Giacomo Gastaldi

**Affiliations:** ^1^ Institute of Psychology and Education, University of Neuchatel, Neuchâtel, Switzerland; ^2^ Department of Medicine, Diabetology Unit, Geneva University Hospital, Geneva, Switzerland

**Keywords:** type 1 diabetes, diabetes self-management, learning process, psychology, remote delivery, diabetes self-management education

## Abstract

Type 1 diabetes management is a highly demanding task that largely falls on people with diabetes, their family, and their peers. Diabetes self-management education and support aim at increasing knowledge, skills, and confidence to take appropriate diabetes management decisions. The current evidence shows that efficient diabetes self-management relies on person-centered interventions and a team of pluri-disciplinary educators with expertise in diabetes care and education. The irruption of the COVID-19 pandemic has increased diabetes burden and the need to offer remote diabetes self-management education services. The present article offers a perspective about expectations and quality issues related to the implementation of a remote version of the FIT course, a validated structured diabetes management educational program.

## Introduction

Type 1 diabetes (T1D) management is a highly demanding task that largely falls on people with diabetes (PWD), their family, and their peers. Diabetes Self-Management Education and Support (DSMES) aims at constructing with PWD the knowledge, skills, and confidence to take appropriate diabetes management decisions (1). Despite evident progress in diabetes technology (D&T), science of psychology and approaches to diabetes management achievement of glycemic control remain suboptimal for a majority of PWD. The irruption of the COVID-19 pandemic has even increased diabetes burden (2). In case of preexisting T1D and coronavirus disease, PWD have a higher risk of diabetic ketoacidosis, hospitalization (3), and illness severity (4).

In 2021, while the world is still marching along through the COVID-19 pandemic, the first guidelines for T1D in adults have been jointly published by the American and European Diabetes Associations (EASD/ADA) (5). This consensus report addressed the numerous topics to consider for T1D management including the role of access to DSMES services and the potential of digital health. Indeed, COVID time has forced the widespread adoption of remote visits (videoconference or phone-call visits) and suggested a true potential to deliver online education (5).

This article discusses, during the COVID period, the implementation of a remote FIT course and considerations related to its impact on the learning process.

## People Living With T1D Face an Unmet Need for DSMES

Diabetes self-management (DSM) is a complex process involving the individual capacity to transform a sum of knowledge, emotions, and daily experiences over diabetes into practical skills. The current evidence shows that efficient DSM relies on person-centered interventions and the DSMES team with deep expertise in diabetes care and education. Therefore, professional educators involved in DSMES need continuing education about pharmacotherapy, D&T, educational strategies, and psychosocial issues, among others (1, 5).

The FIT course training stands for Functional Insulin Therapy and is originally an Austrian-structured DSMES program that has been early implemented in Switzerland and extensively researched in the UK under the acronym DAFNE (6–8). The training permits to experiment the benefits of being more precise with insulin dosage and to gain flexibility in daily life. Outcome studies have shown a positive impact on glycemic control, on frequency of severe hypoglycemia, and on quality of life (9). The participants are trained in groups of 8–10 people to gain skills on a set of predetermined topics as daily insulin adaptations, interpretation of glucose measures, carb counting, food choices, and physical exercise (10). Follow up data show that the positive effect is limited to 18 months (11).

At the Geneva University Hospital (HUG), the FIT course is based on a written curriculum of seven once-weekly sessions of 2 h according to predetermined topics. Our team has observed that the participants also bring up other topics extremely related to their own experience with diabetes, their bodies, and the action of insulin. These topics constitute a set of daily knowledge that mostly fit the form of the educators’ predetermined curriculum. These themes emerge from the group interactional dynamic (educators–participants and participants–participants) and have an important function in the psychology of learning. They create local connections between educators’ explanations, personal experience, and peer experience. Indeed, these local topics are related to the predetermined topics but oriented by the personal point of view of the participants, highlighting their daily experience of DSM. When participants address different topics but in line with the focus, the boundaries between these and the predetermined one widen their understanding of the topics by increasing their space of appropriation (12, 13). Indeed, in the diabetes context, our team has observed that generating and validating local topics is determinant for the understanding of knowledge, emotions, and involvement in the group dynamic (local effect). Local topics also promote the learning processes related to interoception and therefore health behaviors (14, 15). FIT courses are mainly about learning from experience. The guided exercises are designed to help participants adapt their diabetes management by trying new things, as well as gain a new understanding of their emotions and self-awareness.

### Considerations for Implementing a Remote FIT Course in the Context of COVID-19

Since March 2020, the usual face-to-face FIT courses have been cancelled to reduce the virus transmission and limit the risk associated with coronavirus disease. Therefore, our team, at the Geneva University Hospital (HUG), has implemented a remote FIT training in French.

### Remote FIT Course of Similar Design

All the potential participants were contacted by phone or email and offer to either choose an online version or postpone their attendance to the FIT course. They all accepted to attend the remote version. The timetable and the schedule of the sessions were maintained as planned from 19th of March to the 7th of June 2020. The participants joined the remote FIT course through a webinar link (Zoom^®^) with an access code to attend the training. Ten out of eleven attended the first course, and three participants missed one or two sessions due to familiar, medical, or working reasons. In comparison with the face-to-face FIT course, the attendance was in line with what is usually observed.

The remote FIT course was led by the same experienced multidisciplinary team of educators (a diabetologist, a nurse, and a dietitian). They were in the same room respecting the institutional HUG COVID-19 guidelines (distance between the educators, wearing masks, hands washing).

In terms of contents, all the topics usually covered in the FIT course have been maintained within one exception. The guided exercises targeting carb counting had to be adapted to fit the online service. Educators were able to discuss all topics related to insulin management, its desirable and undesirable effects, hypoglycemia management, food handling, and physical activity.

### Observations of the Geneva Educators’ Team

The first consideration after delivering the remote FIT course was its feasibility. None of the participants expressed difficulties to get connected. In terms of setting, the participants were able to express themselves in turn without difficulties. Speaking time was well distributed within the group. However, in the context of an online FIT course delivered *via* a Zoom^®^ webinar, communication has several specifics as the following:

- There were less overlapping discourses.
- Speech distribution was less flexible (participant–participant; participant–educator; educator–participant) and mainly driven by educators.
- Thus, the organization of turn-takings was less spontaneous with limited participant speech self-select.
- The visibility and accessibility of the participant’s D&T devices (pomp, CGMS, etc.) were limited.
- Some participants had difficulties to find a space with enough intimacy (no camera, noisy outdoor).

In terms of the content, new difficulties and needs have emerged with the online setting. Even though the same curriculum was almost entirely maintained, few adaptations had to be made as the following:

- The participants could not be exposed to the carb counting guided exercise in a real setting (food and dishes) and were asked to evaluate it through the webcam.
- For similar reasons, evaluation of food labels was simplified.

### What Need to Be Done?

For the FIT educators, the remote setting apparently facilitated the process of explanation and made it easier to organize participants’ speech. Nevertheless, these treats can lead educators to feel the need to talk more than participants while their role is to ensure that the floor must be equally distributed. The most important impact of this situation is related to the participants’ discourse construction and the undeniable reduction of the emergence of local topics. Indeed, this kind of communication is less conducive to personal experience discourses, which makes invisible any form of links between the predetermined curriculum and daily diabetes experiences.

In the wake of these observations, educators’ tasks are to maintain a spontaneous interaction between participants. Educators should ensure enough time to the participants for personal experiences introducing it through opened questions about how participants understand some information and how they see it in their daily life.

Opening the interaction for personal experiences, educators ensure the emergence of local topics by the interactional work of the participants. They can stimulate it by introducing to others opened questions and exploring group understandings. This is an important way to make visible the group comprehension about the curriculum content or even misconception about technology acceptance and access.

Remote FIT courses imply for the participants to introduce another sort of intimacy given that PWD are mostly at home during the group sessions. Even though being home can be considered as a great convenience in terms of time saving and location, there are at least two elements to discuss: are the participants assured to have enough intimacy? What about having relatives around? These are important psychological issues, especially when diabetes is a conflictual element in the familial environment.

## Discussion

The irruption of the COVID-19 pandemic and the repeated worldwide containments have forced an abrupt widespread adoption of digital health solutions to deliver DSMES services. Remote FIT course is feasible and has been well accepted by the participants at Geneva University Hospital.

The implementation of the remote FIT course required minor adjustments from our team in terms of setting and content. Thus, the offer will certainly be maintained. In contrast, to preserve the interactional quality between participants and educators, which is critical on the learning process, formal aspects need to be evaluated. According to our expertise, it seems crucial that educators keep their focus on the framework to ensure enough turn-taking of the participants’ speech to support local topic production. Otherwise, remote FIT delivery may lose some of its ability to promote learning by experience through sharing.

There is a need to develop French-language digital health solutions to facilitate the implementation and accessibility of DSMES services. Further research and development are needed to expand the accessibility of DSMES services. The use of technology is promising, but not without guaranteeing the quality of education.

## Data Availability statement

The original contributions presented in the study are included in the article/supplementary material. Further inquiries can be directed to the corresponding authors.

## Author Contributions

All authors contributed to the article and approved the submitted version.

## Conflict of Interest

The authors declare that the research was conducted in the absence of any commercial or financial relationships that could be construed as a potential conflict of interest.

## Publisher’s Note

All claims expressed in this article are solely those of the authors and do not necessarily represent those of their affiliated organizations, or those of the publisher, the editors and the reviewers. Any product that may be evaluated in this article, or claim that may be made by its manufacturer, is not guaranteed or endorsed by the publisher.
